# The Occurrence of Postconflict Skills in Captive Immature Chimpanzees (*Pan troglodytes*)

**DOI:** 10.1007/s10764-016-9893-1

**Published:** 2016-02-22

**Authors:** Samina H. Farooqi, Nicola F. Koyama

**Affiliations:** Research Centre in Evolutionary Anthropology & Palaeoecology, School of Natural Sciences & Psychology, Liverpool John Moores University, Liverpool, L3 3AF UK; Animal Care, Applied Sciences, Wirral Met College, Twelve Quays Campus, Morpeth Dock, Shore Road, Birkenhead, Wirral CH41 1AG UK

**Keywords:** Consolation, Infant chimpanzees, Reconciliation, Social competence

## Abstract

Conflict management strategies can reduce costs of aggressive competition in group-living animals. Postconflict behaviors such as reconciliation and third-party postconflict affiliation are widely accepted as social skills in primates and have been demonstrated in many species. Although immature primates possess a repertoire of species-specific behaviors, it is thought that they gradually develop appropriate social skills throughout prolonged juvenility to establish and maintain complex social relationships within their group. We examined the occurrence of postconflict skills in five immature chimpanzees (*Pan troglodytes*) over 15 mo, focusing on interactions that were not with the subject’s mother. We observed reconciliation, with conciliatory tendencies comparable to adults, and provide the first evidence that captive immature chimpanzees commonly reconciled using social play. However, immatures were not more likely to reconcile valuable than nonvaluable relationships. We also observed third party postconflict affiliation although at a lower level than reported for adults. Our results provide evidence for postconflict skills in immature chimpanzees but the lack of higher conciliatory tendency with valuable partners and low occurrence of third-party affiliation indicates extended juvenility may be required refine these skills. Further work is needed to investigate whether these behaviors have the same function and effectiveness as those found in adults.

## Introduction

Living in a group inevitably involves competition for limited resources between conspecifics. Investing in social relationships is one way for animals to increase their competitive ability and/or reduce the costs of competition. Where competition takes the form of aggressive conflict, costs can include risk of injury, increased stress, and potential damage to social relationships (Aureli *et al*. 2002; Aureli and de Waal 2000). Conflict management strategies provide one way to alleviate these negative consequences. They include friendly postconflict reunion between former opponents, known as reconciliation, and third-party postconflict affiliation between a bystander and victim of aggression, that has been referred to functionally as consolation (De Waal and van Roosmalen 1979). Such behavioral strategies are viewed as part of a suite of social skills (Kempes *et al*. 2009) in primates that are learned during a “socialization period” in infancy (Bekoff 2001) and develop through a period of extended primate juvenility (de Waal 1989; Goodall 1986; Joffe 1997; Lonsdorf and Ross 2012; Pagel and Harvey 1993; Poirier and Smith 1974; Watts and Pusey 2002). For example, the play of orphaned chimpanzee (*Pan troglodytes*) juveniles was more likely to result in aggression than the play of mother-reared juveniles, strengthening the idea that social skills are learned in early infancy (Van Leeuwen *et al*. 2014).

Since it was first documented in chimpanzees (de Waal and van Roosmalen 1979), studies have documented the occurrence of reconciliation in >30 primate species (Aureli *et al*. 2002) across strepsirrhines (Verreaux’s sifaka, *Propithecus verreauxi*: Palagi *et al*. 2008); monkeys, e.g., bonnet macaques (*Macaca radiate*: Cooper *et al*. 2007; white-faced capuchins (*Cebus capucinus*: Leca *et al*. 2002); and apes, e.g., bonobos (*Pan paniscus*: Clay and de Waal 2014 and mountain gorillas, *Gorilla gorilla beringei*: Watts 1995) as well as a few nonprimate species such as corvids, e.g., ravens (*Corvus corax*: Fraser and Bugnyar 2011); domestic dogs (*Canis familiaris*: Cools *et al*. 2008); bottlenose dolphins (*Tursiops truncates*: Yamamoto *et al*. 2015); domestic goats (*Capra hircus*: Schino 1998); horses (*Equus caballus*: Cozzi *et al*. 2010); spotted hyenas (*Crocuta crocuta*: Wahaj *et al*. 2001); and wolves (*Canis lupus*: Baan *et al*. 2014). Reconciliation has been shown to reduce the likelihood of renewed aggression and postconflict stress (Aureli and van Schaik 1991; Cooper *et al*. 2007; Das 2000; Koski *et al*. 2007b; Fraser *et al*. 2008; Watts *et al*. 2000) and restore relationships (Cords 1992; Koyama 2001), in particular, relationships that are important to individuals, such as friendships and coalitions (the “valuable relationship hypothesis”: de Waal and Aureli 1997). Much less research has addressed reconciliation by immature primates (long-tailed macaques, *Macaca fascicularis*: Cords 1988; Cords and Aureli 1993; stumptailed macaques: *M. arctoides* and rhesus macaques, *M. mulatta*: de Waal and Johanowicz 1993; Japanese macaques, *M. fuscata*: Kutsukake and Castles 2001, Schino *et al.*^1998^; brown capuchins, *Cebus apella*: Weaver and de Waal 2000, 2003; bonobos: Clay and de Waal 2013a). These studies, mostly in monkeys, have reported that juveniles are able to reconcile their conflicts. Unrelated juvenile long tailed macaques were more likely to reconcile than related pairs (Cords 1988; Cords and Aureli 1993) and juvenile females were more likely to reconcile with unrelated adult female opponents than juvenile males (Cords and Aureli 1993). These findings may relate to the value of the relationship with these partners; however, no studies have yet tested the valuable relationship hypothesis in immature primates using measures of affiliation to determine relationship value. Besides reconciliation, other conflict management mechanisms can co-occur, for example, victims of aggression can receive solicited or unsolicited friendly contact from a third party or bystander not involved in the conflict (Verbeek and de Waal 1997). Such contact potentially functions as consolation (de Waal and Aureli 1996; Fraser *et al*. 2008) and has been reported for great apes (Clay and de Waal 2013a, b; Cordoni *et al*. 2006; Fraser and Aureli 2008). However, reports for monkeys have been variable as studies have reported an absence of third-party postconflict affiliation in some macaque species (de Waal and Aureli 1996), an absence of functional consolation in stumptailed macaques (Call *et al*. 2002) and mandrills (*Mandrillus sphinx*: Schino and Marini 2012) but the occurrence of consolation (distress alleviation and preferential direction toward friends) in Tonkean macaques (*M. tonkeana*: Palagi *et al*. 2014).

Chimpanzees are highly social animals with complex social behavior related to the fission–fusion structure of their society (Boesch and Boesch-Achermann 2000). Living in complex social groups, chimpanzees require cognitive and behavioral skills to successfully maintain cooperative relationships (Boesch 2003; Goodall 1986; Muller and Mitani 2005). Given that chimpanzees do not become sexually mature until the age of 9 yr, they have an extended period in which to acquire these skills such as reconciliation and third-party postconflict affiliation. Both in the wild and in captivity, many studies have documented reconciliation (Arnold and Whiten 2001; Baker and Smuts 1994; de Waal and Aureli 1996; de Waal and van Roosmalen 1979; Fraser and Aureli 2008; Fraser *et al*. 2010; Fuentes *et al*. 2002; Koski *et al*. 2007a; Preuschoft *et al*. 2002; Wittig and Boesch 2003, 2005) and third-party affiliation (de Waal and van Roosmalen 1979; Fraser and Aureli 2008; Koski and Sterck 2007, 2009; Palagi *et al*. 2006b; Romero and de Waal 2010; Romero *et al*. 2010; Wittig and Boesch 2003) in adult chimpanzees. No studies have yet investigated the occurrence of postconflict behavior in immature chimpanzees.

Determining which postconflict skills chimpanzees have acquired by the beginning of juvenility is important to understand the process of social skill acquisition. We investigated postconflict behavior in immature chimpanzees and excluded mothers as social partners in our analyses as we were interested in the occurrence of postconflict behavior as a means to contact other group members. We hypothesized that reconciliation occurs in immature chimpanzees (Hypothesis 1a). Given the extended period of juvenility for the development of social skills in chimpanzees, we predict that immature chimpanzees (up to 7 yr) will not have acquired conciliatory tendencies comparable to those previously reported for adults. As the immatures were likely to have established play relationships with other immatures in the group, the greater compatibility or accessibility (Cords and Aureli 2000) with immature opponents should facilitate postconflict affiliation. Thus, opponent’s age category (immature/adult) should affect conciliatory tendency (Hypothesis 1b). Further, if reconciliation functions to repair bonds that have been strained during the previous conflict, it should be most predictable among individuals that have a valuable relationship (de Waal and Aureli 1997; de Waal and Yoshihara 1983; Kappeler and van Schaik 1992). We, therefore, tested the hypothesis that relationship quality affects conciliatory tendency (Hypothesis 1c). The first affiliative postconflict contact can take many forms in adult chimpanzees, for example, mouth-to-mouth kiss, sitting in contact or brief touch, with one of the most common being grooming (Arnold and Whiten 2001; de Waal and van Roosmalen 1979). In a previous report on the adults in our study group, the most commonly occurring reconciliatory behavior was grooming (Fraser and Aureli 2008). However, grooming is used less frequently by infants and juveniles to contact group members other than their mother/siblings (Goodall 1986; Nishida 1988). Young chimpanzees are more likely to use play behavior to acquire a central position in the group from which they can form affiliative relationships (Shimada and Sueur 2014). Play, rather than grooming, was also used by young chimpanzees to contact other group members during a period of greater tension (Palagi *et al*. 2006b). We therefore hypothesized that immatures would use specific behaviors to reconcile (Hypothesis 1d) and predict that immature chimpanzees will use social play rather than grooming to reconcile with former opponents.

Given that juvenile primates are capable of postconflict reconciliation, we tested the hypothesis that immature chimpanzees engaged in other postconflict behavior, such as third-party contact with the recipient of aggression (Hypothesis 2a). Finally, we were interested in the co-occurrence of postconflict skills and hypothesized that the tendency to perform reconciliation would be related to the tendency to perform third-party affiliation with a recipient of aggression (Hypothesis 2b).

## Methods

### Subjects and Housing

The group of chimpanzees housed in Chester Zoo, UK, comprised 29 related and unrelated individuals (5 adult and 1 adolescent male, 18 adult females, 5 infants/juveniles). Goodall (1986) defined infancy as <5 yr and the juvenile period from 5 to 7 yr although recent studies have grouped immatures as individuals younger than 12 yr (Markham *et al*. 2015). At the start of our study, four immatures were infants <5 yr old and one was a juvenile. By the end of our study, two of the infants were 60 mo and entering juvenility. For simplicity, we refer to the focal subjects as immatures (Table I) throughout. They were all born and reared by their mothers in the zoo. Four of the immatures had relatives in the group, excluding their mothers, totalling seven dyads (relatedness coefficient *r* = 0.25 for two dyads and 0.125 for five dyads). Relatives were all adults. Group composition did not change during the study period with the exception of the birth of a female infant, Tina, in February 2009 and the death of a female infant, Rhiannon, in June 2008. All interactions with Rhiannon were excluded from the dataset.

**Table I:** The sex and age range (months) from the start to the end of the study (September 2008–November 2009) of the five immature chimpanzees in Chester Zoo, UK

Subjects	Sex	Age range (mo)
Dona	F	40–55
Carlos	M	42–57
Dido	F	45–60
Frankie	F	45–60
Eric	M	63–78

The chimpanzee enclosure at Chester Zoo consisted of an outdoor grassed island, *ca*. 2000 m^2^, separated from the public by a 3-m moat and a 143 m^2^ dome-shaped indoor enclosure. The outdoor enclosure was enriched with trees, shrubs, rocks, logs, hammocks, and climbing structures. The indoor area had a 9-m-high iron frame with platforms, ropes, and nets strung from the frame and walls. The chimpanzees were fed two to three times a day and had *ad libitum* access to water both inside and outside. The observer was able to move easily between the two enclosures to maintain visibility of the subjects.

### Data Collection

S. Farooqi collected all data over 15 mo (September 2008–November 2009), recording the time (in s) immatures engaged in play and grooming during 15-min continuous focal animal samples (Altmann 1974) using Observer 5.0 (XT Noldus). We ensured focal samples were selected in random order and for approximately equal amounts of time and collected a total of 706 focal samples (mean ± SD = 141.2 ± 2.9). Following de Waal and Yoshihara (1983), we recorded aggressive conflicts, noting the identities of the victim (the individual that first received aggression) and the main aggressor (the individual that attacked with the most intense aggression). We began a 5-min postconflict (PC) observation immediately after the conflict ended, noting the time/date and continuously recorded all social interactions using a dictaphone: proximity, kiss, play, grooming received, grooming given, mutual grooming, and sitting in contact (Table II). This allowed us to determine the timing of behavioral events. If the conflict was renewed within 2 min of the start of the PC we abandoned the observation and restarted once the renewed conflict ceased. If the second conflict was more aggressive, we recorded the PC after this and ignored the initial conflict.

**Table II:** Definitions of the behavioral categories for the chimpanzees at Chester Zoo, UK from September 2008 to November 2009

Behaviors	Definition
Groom given	Picking through and/or slow brushing aside of the fur of another individual with one or more hands.
Groom received	Another individual(s) picks through and/or slowly brushes aside the fur of the focal individual with one or more hands.
Groom mutual	Two chimpanzees pick through and/or slowly brush aside the fur of each other simultaneously.
Sitting in contact	Huddling with another individual or with a significant portion of body contact. Includes embraces with open arms.
Proximity	The focal individual is within an arm’s length from another individual or individuals, no touching of body. parts..
Aggression	A threat, charging display, chase, grasp, push, or throwing of an object and any contact with another involving kick, hit, stamp, drag, tug hair, bite, or scratch.
Play	Relaxed slow movements of single individual; lying in hammock; playing with ropes, rags, and blankets; somersaulting or tickling or slow grappling between two or more individuals. No running or chasing. Behavioral elements of play including fast grappling, tumbling, wrestling, moving across circles, tackling, stomping, slapping, dragging by limbs, and slamming on the ground.
Kiss	Mouth-to-mouth contact.

We recorded a matched-control (MC) observation the following, or next possible, day at the same time and under similar conditions, i.e., when opponents were visible to each other (Koski *et al*. 2007a) but when there had been no agonistic interaction between opponents for ≥15 min. If these conditions were not met, we postponed the MC until the next day or up to a maximum of 1 week. If we could not obtain a matched control within 1 week, we discarded the corresponding PC.

We also applied the PC–MC method to record third-party affiliative contact (Call *et al*. 2002) from these conflicts involving at least one immature. We considered contact when an immature third party initiated affiliative contact with a recipient of aggression (also known as true consolation: Verbeek and de Waal 1997). Third-party affiliative contact was considered “solicited” (Verbeek and de Waal 1997) when the recipient approached or stretched a hand toward the third party before the interaction (Fraser and Aureli 2008).

### Data Analysis

Our sample included an older male infant that transitioned to juvenility during the study. We checked that his behavior was not consistently higher than the other immatures so that we could include him in our analysis. We analyzed differences using paired *t*-tests (df = 4) and where necessary transformed data to meet assumptions of normality. When comparing the proportion of dispersed pairs (all zeros) with attracted pairs, we used a one-sample *t*-test. Performing nonparametric statistics did not alter the significance of the results. Where appropriate, we report mean (±SD) values in the text. We performed statistical analyses using SPSS 20 and all tests were two-tailed with the significance level set at *P* < 0.05.

We collected a total of 176 PC observations, excluding conflicts with mothers, of which seven were discarded because no MCs were obtained within the following 7 days. A mean number of 33.8 ± 18.3 PC–MC pairs per focal subject were recorded from 61 conflicts between immatures and 108 conflicts between immatures and adults. Each PC–MC pair was labeled: attracted, if the first affiliative interaction between opponents occurred earlier, or only in the PC relative to the MC; dispersed, if it occurred earlier or only in the MC; and neutral, if there was no affiliative interaction between the opponents in either observation or if it occurred at the same time in both the PC and the MC. To test whether immatures reconciled their conflicts (Hypothesis 1a), we compared the proportion of attracted and dispersed pairs (Fraser and Aureli 2008). We tested this for all PC–MC pairs and then separated conflicts between immatures and those between immatures and adults to test whether immatures reconciled conflicts amongst themselves, as well as those with adults. To test whether the occurrence of reconciliation was not due to one or two immatures, we calculated the corrected conciliatory tendency that controls for baseline levels of affiliation (Veenema *et al*. 1994) for each individual as 100 × ([number of attracted pairs – number of dispersed pairs]/total PC–MC pairs). We then tested for a difference between individuals’ corrected conciliatory tendency with other immatures and with adults (Hypothesis 1b).

We used the adult–immature conflicts only (*N* = 108) and excluded the adult-initiated reconciliations (*N* = 101) to test the effects of kinship and valuable relationships on corrected conciliatory tendencies (Hypothesis 1c). As there were only four PC–MC pairs for two kin dyads we could not compare corrected conciliatory tendencies between kin and nonkin. To test the valuable relationship hypothesis, we excluded PCs between kin (*N* = 97, mean ± SD number of PCs per immature = 19.4 ± 11.8 and mean number of opponents per immature = 9 ± 3.8) and for immatures, compared the corrected conciliatory tendency with their valuable partners to the corrected conciliatory tendency with their nonvaluable partners (Hypothesis 1c). We defined valuable partners as those that were grooming or play partners. Given that this is a captive group living in close quarters, proximity relationships may not accurately reflect relationship quality. Owing to the low occurrence of grooming (9 of the 44 PC adult–immature dyads groomed at some point but at low rates), we labeled any adult with which an immature exchanged grooming as a grooming partner. As play is one of the most important social interactions for immatures to contact other group members, we incorporated play behavior. Thirty-two of the 44 immature–adult PC dyads played with each other at some point. We therefore defined valuable play partners as those that played above an individual’s mean and nonvaluable play partners as those that never groomed or played above an individual’s mean.

We tested the most commonly used reconciliatory behaviors to see whether they were more likely to occur in the PC than in the MC. To assess whether grooming or play were used preferentially as a reconciliatory behavior by immatures, we compared the proportions of first PC contacts that were play with those that were grooming (Hypothesis 1d).

We investigated the occurrence of unsolicited and solicited third-party affiliative contact separately (Hypothesis 2a). For unsolicited contact we identified where each immature acted as a third party initiating PC affiliative contact with the recipient of aggression and compared this to the occurrence and timing of affiliative contact between the same third party and recipient of aggression in the MC. We then labeled PC–MC pairs as attracted, if contact occurred only in the PC or earlier in the PC than in the MC; dispersed, if it occurred only in the MC or earlier in the MC than in the PC; and neutral, if there was no affiliative interaction in either the PC or the MC, or it occurred at the same time in both. We then tested for a difference between the proportion of attracted and dispersed pairs to determine the occurrence of third-party PC affiliation. Solicited third-party PC affiliative contact occurred too rarely to be analyzed. For comparison with previous analyses, we calculated individual triadic conciliatory tendency (Call *et al*. 2002) for each immature as a measure of third party postconflict affiliation received: 100 × ([number of attracted pairs – number of dispersed pairs]/total PC–MC pairs). Triadic conciliatory tendency is an index that is calculated for individual victims and reflects contact received or solicited. Thus to examine third-party affiliative contact offered by immatures we present the mean frequency of contact given by immature third parties and give the proportions offered to adult and immature victims. We compared the latency to first affiliative contact between the victim and third party in PC–MC observation in a 5-min time window. To investigate whether corrected conciliatory tendency and triadic conciliatory tendency co-occurred, we used a Pearson correlation to test for a relationship (Hypothesis 2b).

## Results

**Fig. 1:**
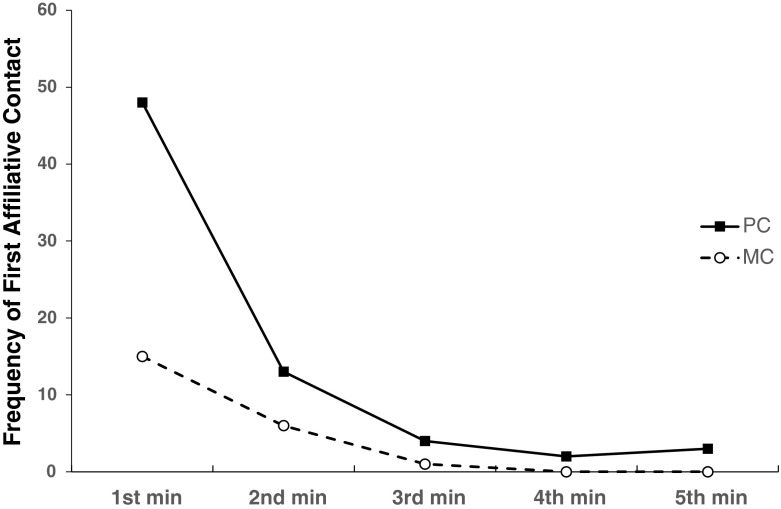
The frequency of first affiliative contact between former chimpanzee opponents during each minute of the postconflict (PC) and matched control (MC) observations collected at Chester Zoo, UK from September 2008 to November 2009.

Following the PC–MC method, we found the proportion of attracted pairs (0.43 ± 0.07) was significantly higher than the proportion of dispersed pairs (0.12 ± 0.03, *t* = 8.2, *P* < 0.001), indicating that the majority of affiliative contacts between the opponents occurred earlier in the PC than in the MC and demonstrating the occurrence of reconciliation (Hypothesis 1a). Former opponents were more likely to make affiliative contact in the first minute of observation (Fig. 1). Overall, the mean group corrected conciliatory tendency for the immature chimpanzees was 31.4 % ± 4.3 (Table III). When we selected only conflicts between immatures (*N* = 61) we found that the proportion of attracted pairs (0.53 ± 0.12) remained higher than the proportion of dispersed pairs (0.18 ± 0.05, *t* = 4.89, *P* = 0.008), demonstrating the occurrence of reconciliation between immatures. Likewise for conflicts between immatures and adults (*N* = 108), the proportion of attracted pairs (0.33 ± 0.09) was higher than the proportion of dispersed pairs (0.07 ± 0.06, *t* = 8.33, *P* < 0.001).

**Table III:** Individual corrected conciliatory tendencies (CCT) and triadic contact tendencies (TCT) for each immature chimpanzee over the course of PC data collection at Chester Zoo, UK from September 2008 to November 2009

Immatures	CCT (%)	TCT (%)
Dona	26.3	10.5
Carlos	25.4	11.3
Dido	46.2	7.7
Frankie	28.9	8.7
Eric	30	11.5
Mean (±SD)	31.4 ± 8.5	10.0 ± 1.7

Within the 70 attracted pairs, affiliative contact was initiated by immatures in 63 PCs and by adults in only 7 PCs. Excluding the adult-initiated contacts, the proportion of attracted pairs (0.27 ± 0.15) remained higher than the proportion of dispersed pairs (0.07 ± 0.06, *t* = 3.12, *P* = 0.036). The mean individual corrected conciliatory tendency for immature–immature conflicts (36 % ± 13.8) did not differ from that for immature–adult conflicts (26 % ± 3.9, *t* = 1.02, *P* = 0.4, Hypothesis 1b). Neither did we find any difference in mean individual corrected conciliatory tendencies with valuable partners (39 % ± 22.5) and nonvaluable partners (24 % ± 18.3, *t* = 2.0, *P* = 0.19) as only three (Dona and Carlos, the youngest and Eric, the oldest) immatures had higher corrected conciliatory tendencies for valuable than nonvaluable partners.

The most frequently occurring reconciliatory behaviors were social play (37 %), arm’s length proximity (30 %), and sit in contact including embrace (24 %). Play was significantly more likely to occur after a conflict compared to the control period (play: *t* = 4.5, *P* < 0.02); however, proximity (*t* = 1.6, *P* > 0.1) and sit in contact including embrace (*t* = 2.1, *P* > 0.1) were not more likely to occur relative to the control. Other behaviors used for reconciliation were grooming (6 %), which occurred much less frequently, and kissing, which occurred only twice (3 %). The first case of kissing was after aggression between Carlos and an adult male; Carlos approached the adult and both kissed. In the second case, Eric kissed Dido after he had hit her hard. Immature chimpanzees were significantly more likely to use play rather than grooming as a reconciliatory behavior, supporting our prediction for Hypothesis 1d (*t* = 5.87, *P* = 0.004).

**Fig. 2:**
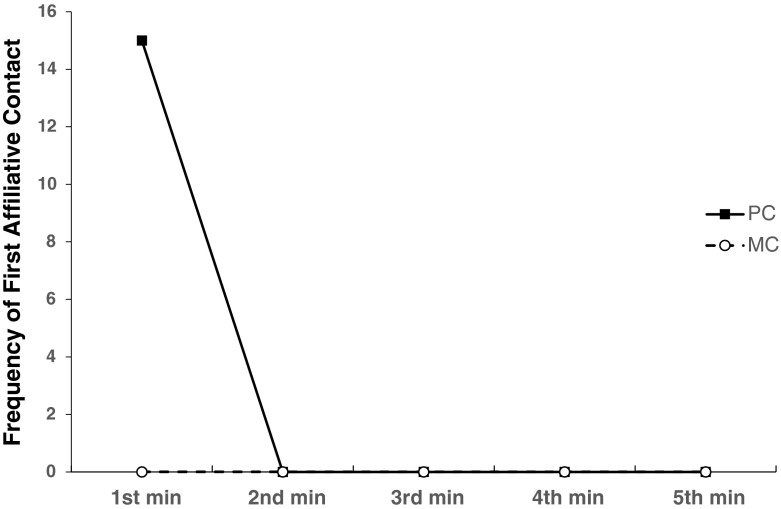
Frequency of first affiliative contact by immature third party to a recipient of aggression in each minute of the postconflict (PC) and matched-control (MC) periods collected at Chester Zoo, UK from September 2008 to November 2009.

Considering third-party postconflict contact, the proportion of attracted pairs (0.1 ± 0.06) was significantly higher than the proportion of dispersed pairs (0.0 ± 0.0), demonstrating the occurrence of third-party postconflict affiliation (*t* = 3.8, *P* < 0.02, Hypothesis 2a). All immatures offered this behavior with the exception of one female immature (mean ± SD frequency given by immatures was 3.8 ± 1.3). Only immature victims were the recipients of third-party postconflict affiliation. As victims of aggression, all immatures received postconflict affiliation from bystanders, and the mean triadic conciliatory tendency for the immatures was 10 % (Table III). The temporal distribution of the frequency of first affiliative contacts from immature third parties to the recipients of aggression showed that all contacts occurred during the first minute of the PC (Fig. 2). Solicited third-party postconflict affiliation occurred too rarely to be analyzed (two cases).

When we tested for an association between triadic conciliatory tendency and corrected conciliatory tendency (Hypothesis 2b) we found no significant association (*r*_5_ = –0.27, *P* = 0.9).

## Discussion

We quantitatively demonstrated the occurrence of reconciliation and third-party postconflict affiliation in immature chimpanzees. As the sample size was small and included four infants and one older infant that became a juvenile within the study period, our findings should be interpreted with caution and await replication; nonetheless, the presence of these behaviors suggests that by the end of infancy and beginning of juvenility (5–6 yr) chimpanzees have acquired postconflict social skills commonly reported in adults (Fraser *et al*. 2010; Preston and de Waal 2002). We did not find evidence for solicited third-party postconflict affiliation.

We found that immature chimpanzees were capable of reconciling their conflicts (corrected conciliatory tendency = 31.4 %) and they did so at a comparable, although lower level to that reported for adults in the same group (corrected conciliatory tendency = 47.5 %) around 18 mo before our study (Fraser *et al*. 2008). Although variable, lower corrected conciliatory tendencies have often been reported for wild (14.4–21.6 %) chimpanzees (Arnold and Whiten 2001; Kutsukake and Castles 2004; Wittig and Boesch 2005) than for captive (21.6–41.2 %) chimpanzees (Fraser *et al*. 2008; Koski *et al*. 2007a; Preuschoft *et al*. 2002; *cf*. Fuentes *et al*. 2002; Webb *et al*. 2014). A higher conciliatory behavior has been associated with particularly tolerant populations (de Waal and van Roosmalen 1979).

Postconflict reconciliation has several functions such as reducing levels of postconflict anxiety (Aureli and van Schaik 1991) and restoring tolerance levels and valuable social relationships damaged by the aggressive conflict (de Waal and Aureli 1997). Our results do not address which of these functions reconciliation fulfils in immatures; for example, we did not collect any measures of postconflict anxiety. In contrast to the finding that same aged dyads (adult–adult and adolescent–adolescent) were more likely to reconcile than mixed-aged dyads (Webb *et al*. 2014), we did not find any difference in the corrected conciliatory tendencies of immatures with their peers or with adults. Neither did we find an effect of relationship value on immatures’ conciliatory tendency. This could suggest that immatures had acquired the behavior of postconflict affiliation with former opponents but not the selectivity in reconciling with valuable partners, perhaps owing to a lack of differentiated relationships at this age. Further research is needed to investigate the functional aspects of reconciliation in immature chimpanzees.

Our study is the first to demonstrate that immature chimpanzees preferentially use social play to reconcile with former opponents. In the study group, adult chimpanzees have been previously reported to most commonly reconcile using grooming behavior (nearly 40 %), with behavioral specificity (de Waal 1993) demonstrated for kiss and embrace (Fraser and Aureli 2008). Grooming was used rarely by immature chimpanzees and their preference for play likely reflects their most common form of social interaction with conspecifics at this age (Bloomsmith *et al*. 1994; Shimada and Sueur 2014), despite the fact that play decreases markedly in late infancy (Lonsdorf *et al*. 2014). Play has also been reported to function in reducing tension and confrontations during stressful situations in chimpanzees and bonobos (Palagi *et al.*2006b; Paquette 1994), which adds to its suitability as a reconciliatory behavior. Kissing occurred only twice, suggesting that these may be adult forms of reconciliatory behavior that further develop during juvenility and adolescence. Unfortunately the frequency of embracing could not be determined, as it had been combined with sitting in contact; however, sitting in contact occurred at a much lower rate than play.

Reconciliation merely requires an ability to recognize individuals and remember past interactions, and a conciliatory disposition (de Waal and van Roosmalen 1979). In contrast, consolation is proposed to be cognitively more demanding as it requires some form of sympathetic concern about another’s state, including attempts to ameliorate another’s state (de Waal and Aureli 1996; de Waal 2008; Preston and de Waal 2002; *cf*. Bolhuis 2015; Puga-Gonzalez *et al*. 2014). Our analysis of third-party postconflict affiliation did not include any measure of stress alleviation in the victim and so we cannot interpret this behavior as consolation; nonetheless we can compare the occurrence of the operational definition with other studies. The mean triadic conciliatory tendency for immatures in our study was 10 %, lower than that reported previously for adults: in the same group (29.4 %: Fraser *et al*. 2008); for other captive groups (16.5 % and 10.8 %: Romero and de Waal 2010; 49.5 %: Palagi *et al*. 2006a); or in the wild (15.1 %: Kutsukake and Castles 2004). However, we restricted our data collection to conflicts involving an immature and did not collect conflicts between adults. This may have biased our analysis to lower values of triadic conciliatory tendency and restricted to whom immatures offered affiliation. Unlike previous authors, we did not find that immatures had high rates of third party postconflict affiliation with both adults and infants/juveniles (Clay and de Waal 2013a), but found that immatures offered third party postconflict affiliation only to other immatures. In another group of captive chimpanzees (Palagi *et al*. 2006a), there was no difference in adult–adult, adult–juvenile, or juvenile–juvenile triadic conciliatory tendencies (juveniles were aged 6–8 yr), suggesting that third-party postconflict affiliation is fully acquired and expressed after 6 yr of age. However, they did not include an analysis of the functional aspect of consolation.

It is possible that third-party postconflict affiliation in immatures may be functionally different from consolation in adults. The benefits of “true” consolation are still debated but possible functions include stress reduction (Fraser *et al*. 2008) and distress alleviation, where contact is more likely between friends than non-friends (Fraser *et al*. 2008; Romero and de Waal 2010). There are different levels of empathy (de Waal 2008), from emotional contagion (being affected by another’s emotional or arousal state), to sympathetic concern (appraisal of another’s situation) and empathic perspective taking. It is possible that consolation in immatures and adults may reflect these different levels. Given that infant chimpanzees (aged 36–54 mo) appear to be capable of instrumental helping, i.e., knowing something about the goal another individual is attempting to achieve as well as the current obstacles to that goal (Warneken and Tomasello 2006), it seems reasonable that they may be capable of recognizing and responding to another’s distress, i.e., sympathetic concern.

Future studies should address whether the variation in individual triadic conciliatory tendency (7.7–11.5 %) reflects stable individual variation or underlying cognitive capacity, ideally by combining experimental and social behavioral data. Consistent individual differences in postconflict behavior have been reported for adult and adolescent chimpanzees, where an individual’s conciliatory tendency was associated with social switching behavior that was indexed by changes or switches in social behavioral state or partner (Webb *et al*. 2014). Further, individual emotion regulation is an important component of social competence (Clay and de Waal 2013b). Juvenile bonobos that were better able to manage their own emotions (faster recovery from self-distress and baseline levels of anxiety-related behavior) showed greater social competence (number of friendships, amount of sustained play, and a composite index of sociality) and were more likely to offer consolation.

Finally, we were interested in whether the expression of reconciliation would be associated with the prevalence of third-party postconflict affiliation. However, we did not find any association between corrected conciliatory tendency and triadic conciliatory tendency across immatures. The lack of any association is difficult to explain, as possible interpretations could relate to our small sample size or point to different, underlying capacities associated with reconciliation, consolation, and general sociability.

In summary, our findings provide data on the occurrence of postconflict behavior in immature chimpanzees. We found that immatures were able to perform reconciliation; however, functional aspects, such as reconciling valuable relationships and postconflict behavioral specificity, were not fully developed. Immatures performed third-party affiliative contact, albeit at lower levels than reported in adults. Our findings support the idea that postconflict skills are refined, gradually, through an extended juvenile period. Further work is needed to explore these postconflict behaviors in immatures and determine whether the expression of these skills is influenced by individual differences and the emergence of underlying cognitive capacities and if they are functionally different from postconflict skills found in adults.
